# Prognostic significance of S100A8-positive immune cells in relation to other immune cell infiltration in pre-invasive and invasive breast cancers

**DOI:** 10.1007/s00262-020-02776-5

**Published:** 2020-11-04

**Authors:** Ji Won Woo, Yul Ri Chung, Milim Kim, Hye Yeon Choi, Soomin Ahn, So Yeon Park

**Affiliations:** 1 Department of Pathology, Seoul National University Bundang Hospital, 82, Gumi-ro 173 Beon-gil, Bundang-gu, Seongnam, Gyeonggi 13620 Republic of Korea; 2 Department of Pathology, Seoul National University College of Medicine, Seoul, Republic of Korea; 3 Pathology Center, Seegene Medical Foundation, Seoul, Republic of Korea

**Keywords:** Breast cancer, Progression, S100A8 protein, Myeloid-derived suppressor cell, Tumor-infiltrating lymphocyte, PD-L1

## Abstract

Myeloid-derived suppressor cells (MDSCs) play an important role in tumor progression through both immunologic and non-immunologic mechanisms. This study was conducted to evaluate the expression of S100A8, a well-known MDSC marker, and the significance of its expression in pre-invasive and invasive breast cancers. S100A8 expression in tumor cells (TCs) and immune cells (ICs) was assessed by immunohistochemistry, and its association with clinicopathologic features and infiltration of other IC subsets including CD4+, CD8+, and FOXP3+ tumor-infiltrating lymphocytes (TILs) and PD-L1+ ICs was evaluated. S100A8 expression in TCs and ICs showed a positive correlation in pre-invasive carcinoma and invasive carcinoma. S100A8+ ICs, but not S100A8+ TCs, were significantly higher in number in invasive carcinoma than in pre-invasive carcinoma. Infiltration of S100A8+ ICs was revealed as a poor prognostic indicator in pre-invasive and invasive carcinomas, especially in hormone receptor-positive subgroup. Infiltration of CD4+, CD8+, and FOXP3+ TIL subsets and PD-L1+ ICs was significantly higher in S100A8+ IC (+) group than in S100A8+ IC (−) group. Combined analyses of IC subset infiltration revealed that infiltration of S100A8+ ICs was associated with poor clinical outcome in the PD-L1+ IC (−), CD8+ TIL-low, and FOXP3+ TIL-low subgroups. In conclusion, S100A8+ ICs seem to undergo a dynamic change during breast cancer progression in association with other IC subset infiltration. The prognostic impact of S100A8+ IC infiltration was greater in less immunogenic tumors.

**Electronic supplementary material:**

The online version of this article (10.1007/s00262-020-02776-5) contains supplementary material, which is available to authorized users.

## Introduction

Tumor immune microenvironment is crucial for tumor development and progression, and it is now considered an important therapeutic target. So far, studies on tumor immunity have mainly focused on tumor-infiltrating lymphocytes (TILs) and their interactions with the tumor. As our understanding of immune microenvironment deepens, many agree that tumor immunity cannot be explained solely by lymphoid cells and that there must be other players involved including myeloid-derived suppressor cells [1].

Myeloid-derived suppressor cells (MDSCs) are known to suppress anti-tumor immunity by various mechanisms such as depleting nutrients required by lymphocytes, generating oxidative stress, interfering with lymphocyte trafficking and viability, activating and expanding regulatory T cell populations, decreasing effector T cell function, and inducing PD-L1 expression [2–4]. Furthermore, MDSCs are involved in tumor progression through non-immune-mediated mechanisms: they stimulate neovascularization by secreting vascular endothelial growth factor (VEGF) and basic fibroblast growth factor (bFGF) [5], and promote tumor invasion and metastasis via production of matrix metalloproteinases (MMP) and chemokines [6, 7].

While the importance of MDSCs in tumor progression cannot be overstated, its clinical significance remains vague due to its phenotypic complexity. MDSCs can be divided into two major subsets which have different functions: polymorphonuclear MDSCs [CD11b^+^ CD14^−^ CD15^+^ (or CD66b^+^)] and monocytic MDSCs (CD11b^+^ CD14^+^ HLA-DR^low/−^ CD15^−^) [8]. Most studies on MDSCs have focused on cells in peripheral lymphoid organs and peripheral blood, mainly due to technical challenges associated with MDSC isolation from tumors. Tumor MDSCs, however, are known to be different from peripheral MDSCs with a stronger immunosuppressive activity [3].

As the phenotypic complexity of MDSCs complicates various analyses, surrogate markers of MDSCs have been developed, a well-known marker of which is S100A8 [8]. Originally, S100A8 was known as a pro-inflammatory danger signal expressed by myeloid origin cells (neutrophil, macrophage and monocyte) in an inflammatory environment, but recent studies have focused on the relationship between MDSCs and S100A8 in tumors [9]. In human cancer, monocytic MDSCs rather than polymorphonuclear MDSCs seem to be the major source of S100A8 although studies on S100A8 production by MDSC subsets have shown great variations by cancer type [9]. S100A8 forms a stable protein complex with S100A9 and works as a heterodimer of S100A8/A9 known as calprotectin. It generates and recruits MDSCs, and it supports an autocrine feedback loop that sustains accumulation of MDSCs in a tumor [10, 11]. S100A8 is known to promote tumor proliferation and migration, and it even forms pre-metastatic niches [12–14].

S100A8 can be expressed in both immune cells (ICs) and tumor cells (TCs). Expression of S100A8 in invasive breast carcinoma has been studied using immunohistochemistry [15–19]. Expression of S100A8 in pre-invasive breast carcinoma, however, has not been studied widely although it is hypothesized that S100A8 exerts its pro-tumorigenic role in pre-invasive cancer as a MDSC-associated molecule [19–21]. Moreover, there have been no studies on the correlation between S100A8+ ICs and TIL subsets or PD-L1+ ICs, despite the possible close relationship with each other considering various immunomodulatory functions of MDSCs.

Thus, in this study, we aimed to evaluate the difference in expression of S100A8 in TCs and ICs between pre-invasive carcinoma and invasive carcinoma of the breast, and we investigated the clinicopathologic significance of its expression. We also evaluated the relationship between S100A8+ ICs and other TIL subsets infiltration including CD4+ , CD8+ , and FOXP3+ TILs and PD-L1+ ICs as well as the prognostic significance of S100A8+ ICs in various immune environments.

## Materials and methods

### Tissue samples

A total of 176 cases of pre-invasive carcinoma and 524 cases of invasive carcinoma of the breast were included in the study. Patients who received surgical resection for primary breast cancer at Seoul National University Bundang Hospital between 2003 and 2011 were included, and the histological slides were retrieved from the archive of the Department of Pathology. Male patients and those with distant metastasis at the time of diagnosis were excluded.

Medical records were reviewed to obtain clinicopathologic data. The following information was recorded for all cases: age, sex, information on neoadjuvant and adjuvant therapy, histologic subtype (by WHO classification), estrogen receptor (ER), progesterone receptor (PR), human epidermal growth factor receptor 2 (HER2), Ki-67, and p53 status. For invasive carcinoma, size of tumor, histologic grade (by Nottingham combined histologic grading system), lymphovascular invasion (LVI), TNM stage (by 7th American Joint Committee on Cancer staging system), and survival data were also collected. Deaths unrelated to breast cancer, such as a traffic accident or an underlying medical condition were separately annotated for analysis of disease-specific survival. For pre-invasive carcinomas, tumor extent, nuclear grade, comedo-type necrosis, and information on recurrence were recorded. The clinicopathologic characteristics of the patients are summarized in Supplementary Tables S1 and S2.

### Tissue microarray construction

Histologic slides from all of the cases were reviewed to determine the area most representative of the tumor. For pre-invasive carcinoma, one to three tissue cores (depending on the extent of the tumor) of 4-mm diameter were arranged in a tissue microarray (TMA) using a trephine apparatus (Superbiochips Laboratories, Seoul, Korea). TMAs of 2-mm diameter with 3 cores per case were constructed for invasive carcinoma (Superbiochips Laboratories).

### Immunohistochemistry and S100A8 scoring

Immunohistochemical staining for S100A8 was performed on TMAs after staining optimization using positive and negative control and serial dilution. Briefly, sections from TMAs were submitted to routine immunohistochemical techniques including deparaffinization and rehydration in graded ethanol. Antigen retrieval was performed by immersing the slides in citrate buffer (pH 6.0) for 30 min in a steamer. Endogenous peroxidase activity was blocked with a 3% H_2_O_2_–methanol solution, and the slides were incubated in 10% normal goat serum for 30 min to prevent non-specific staining. They were then incubated for 1 h at room temperature with an anti-S100A8 antibody (clone EPR3554; 1:2000; Abcam, Cambridge, UK). Thereafter, the sections were incubated with horseradish peroxidase-labeled polymer conjugated with secondary antibodies (DAKO Envision detection kit, Dako, Carpinteria, CA, USA) for 30 min. Diaminobenzidine was used as a chromogen, and the sections were counterstained with Mayer’s hematoxylin.

S100A8 expression was separately evaluated in TCs and ICs by two pathologists (JWW and SYP) blinded to clinicopathologic information. The distinction of S100A8 expression between TCs and ICs was determined by histologic findings of the S100A8+ cells including their location (in the tumor cell nest vs. in the stroma), overall cell morphology (epithelioid vs. monocytoid, stellate or spindle shaped), and nuclear feature (atypical vs. bland-looking). In some case where this distinction was difficult, simultaneous comparison of H&E- and S100A8-stained slides was performed. For TCs, the percentage of positively stained tumor cells was counted regardless of intensity or staining pattern (cytoplasmic, membranous, or nuclear) as a previous study had reported that correlation with various clinicopathologic features was irrespective of the location of positive S100A9 staining [16]. Scoring was done as a continuous variable, and it was dichotomized to either S100A8+ TC-negative or -positive group according to the cutoff value of 0% estimated by receiver operating characteristic (ROC) curve analysis that maximized the sum of sensitivity and specificity in predicting disease-specific death.

S100A8 expression in ICs was evaluated according to preexisting guidelines for PD-L1 expression evaluation in breast cancer [22]. Average percentage of expression was recorded as a proportion of the area occupied by the positively stained ICs of any intensity or morphology in the tumor area including intratumoral and stromal areas. In pre-invasive carcinomas, the stromal compartment was defined as the area of specialized stroma surrounding the involved ducts, or when unclear, as the area surrounding the ducts within 2 high-power fields [23, 24]. Areas with crush artifacts, necrosis, or hyalinization were excluded. Intravascular ICs were not evaluated. The values were recorded as a continuous variable and categorized afterwards to either S100A8+ IC-negative or -positive group with an optimal cutoff value of 5% from ROC curve analysis.

### Data of immune cell subsets

The data of CD4+, CD8+, and FOXP3+ TILs and PD-L1+ ICs were adopted from our previous studies [25, 26] for all of the cases of pre-invasive carcinoma and 307 cases of invasive carcinoma. Immunohistochemical staining had been carried out using the following antibodies: CD4 (clone SP35; ready to use; Dako), CD8 (clone C8/144B; ready to use; Dako), FOXP3 (clone 236A/E7; 1:100; Abcam) and PD-L1 (clone E1L3N; 1:100; Cell Signaling, Danvers, MA, USA). CD4+, CD8+, and FOXP3+ T cells had been counted in intratumoral and stromal areas as absolute numbers per high-power field. Detailed information on the counting method of TILs is described in the previous studies [25, 26]. For this study, CD4+, CD8+, and FOXP3+ TILs were dichotomized into high- and low-infiltration groups using cutoff values obtained by ROC curve analyses. PD-L1+ ICs were considered to be present when at least 1% of the tumor stromal area was occupied by PD-L1+ ICs.

### Evaluation of standard biomarkers and determination of breast cancer subtypes

Expression of the standard biomarkers including ER, PR, HER2, p53, and Ki-67 was evaluated from the surgical resection specimens at the time of diagnosis. Immunohistochemical staining had been carried out on representative tumor sections using the following antibodies: ER (clone SP1; 1:100; LabVision, Fremont, CA, USA), PR (clone PgR 636; 1:70; Dako), HER2 (clone 4B5; ready to use; Ventana Medical Systems, Tuscon, AZ, USA), p53 (clone D07; 1:600; Dako), and Ki-67 (clone MIB-1; 1:250; Dako).

ER and PR were considered positive when 10% or more than 10% of tumor nuclei were stained since it has been suggested that a majority of breast cancers with low (1–10%) ER expression are biologically similar to hormone receptor (HR) negative tumors [27]. HR status was defined as positive when ER and/or PR is positive. HER2 status was defined as positive if HER2 immunohistochemistry showed a score of 3+ or HER2 in situ hybridization showed gene amplification. Ki-67 proliferation index was divided into low and high using a 20% cutoff for invasive carcinoma and a 10% cutoff for pre-invasive carcinoma. For p53, staining in 10% or more of the tumor nuclei was considered positive.

We adopted a simple subtyping method that can be done with standard biomarker profiles according to 2011 St Gallen International Expert Consensus [28]. Each category is defined as follows: luminal A (ER+ and/or PR+, HER2-, Ki-67 < 14%), luminal B (ER+ and/or PR+ , HER2-, Ki-67 ≥ 14%; ER+ and/or PR+ , HER2+), HER2+ (ER−, PR−, HER2+), and triple negative (ER−, PR−, HER2−).

### Statistical analysis

Statistical analyses were performed using SPSS version 25.0 for Windows (IBM Corp., ARMONK, NY, USA). The data of S100A8 expression in TCs and ICs, and CD4+, CD8+, and FOXP3+ TIL counts did not meet the assumption of normality, and thus, non-parametric tests were used. The difference in continuous variables was analyzed by Mann–Whitney *U* test between two groups. For comparison of categorical variables, Chi-square or Fisher’s exact test was used. Spearman’s rank correlation tests were used to assess the correlation between two variables. Survival curves were estimated by Kaplan–Meier method, and the significance was calculated by log-rank test. Cox proportional hazard model was used for multivariate analysis using a backward stepwise selection method. Hazard ratios and their 95% confidence intervals (CI) were calculated for the significant variables. All *p* values were two-sided, and *p* values less than 0.05 were considered statistically significant.

## Results

### *S100A8*+ *tumor cells and immune cells in pre-invasive and invasive carcinoma*

**Fig. 1:**
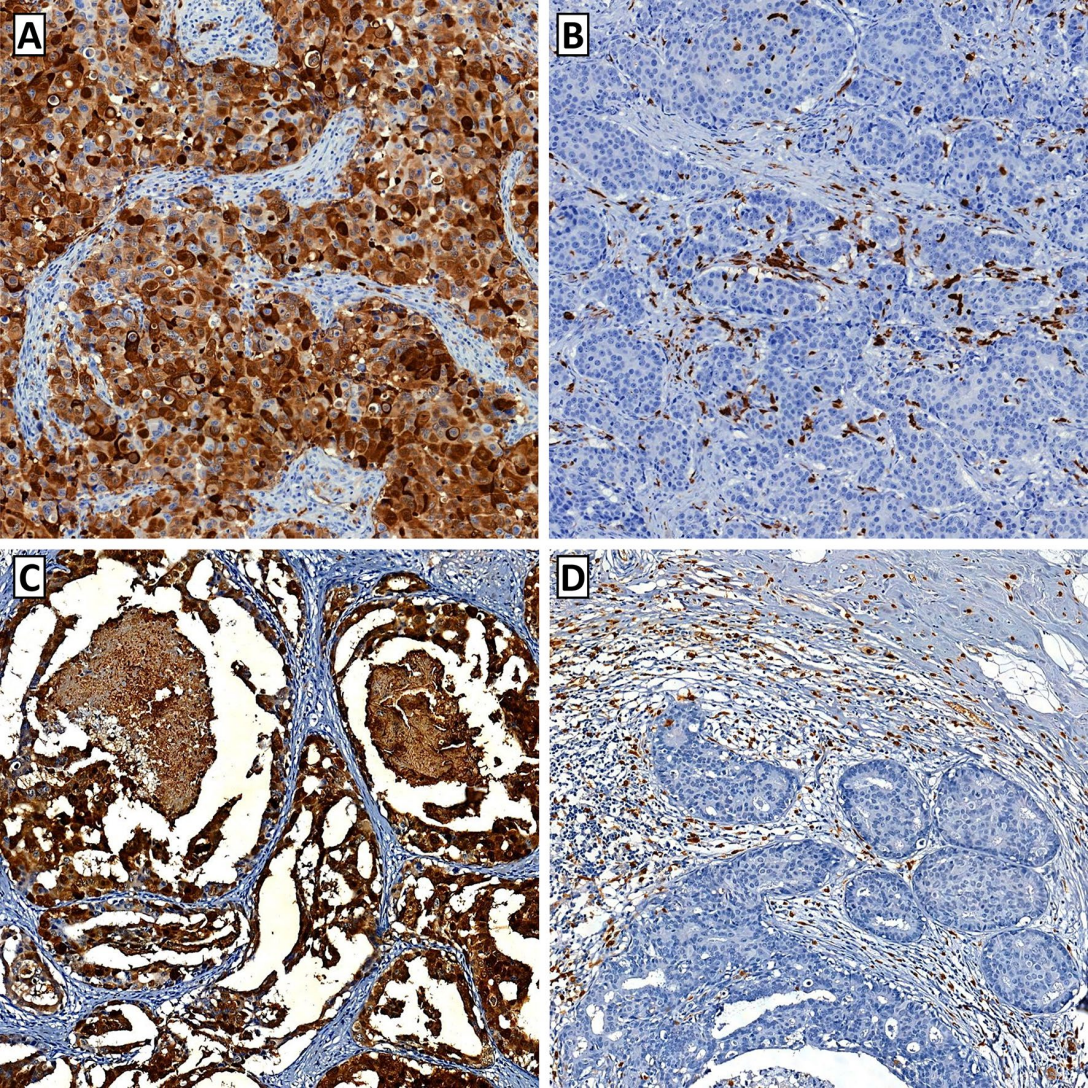
Representative images of S100A8 expression in breast cancer. **a** Tumor cells show strong cytoplasmic expression of S100A8 in an invasive breast carcinoma. S100A8-postive immune cells are rarely found in the stromal area. **b** In this case of invasive breast carcinoma, S100A8-positive immune cells are frequently observed, whereas the tumor cells are totally negative for S100A8. **c** In a case of high-grade ductal carcinoma in situ with comedo-type necrosis, the tumor cells show strong S100A8 expression. **d** In this case of ductal carcinoma in situ, abundant S100A8-positive immune cells are found in association with numerous tumor-infiltrating lymphocytes. The tumor cells are negative for S100A8 expression

**Fig. 2:**
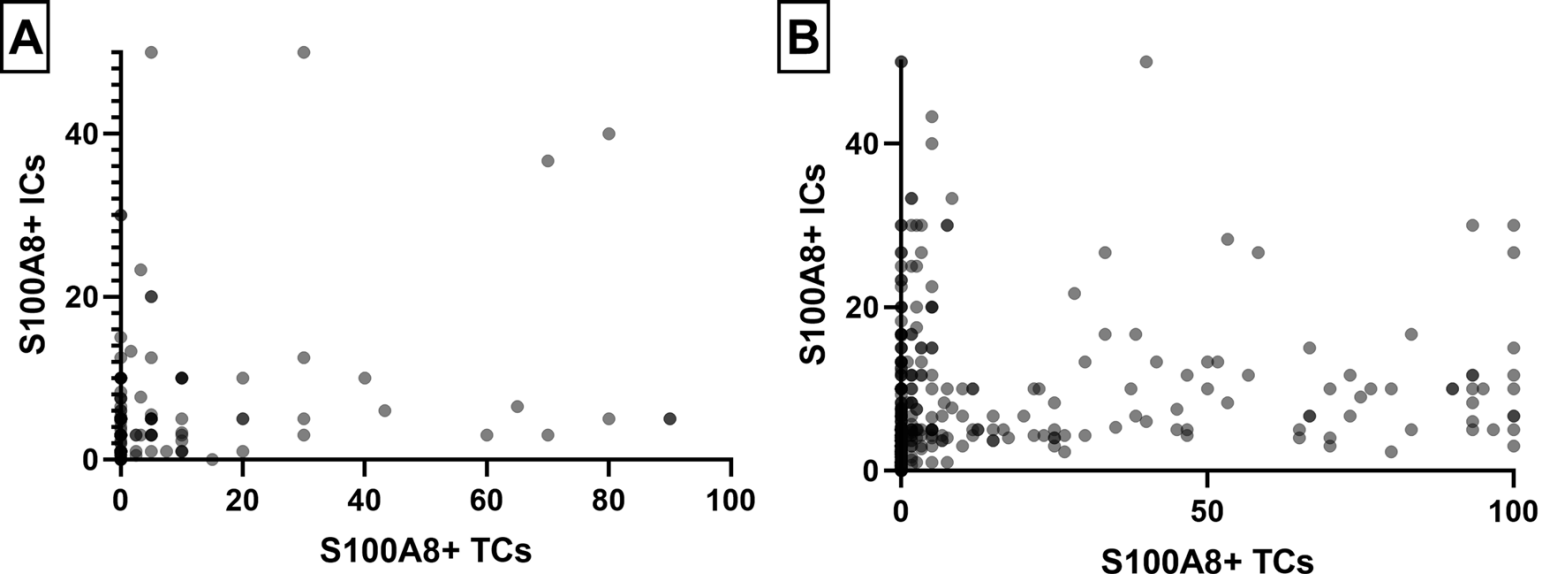
Scatter plots showing the relationship between S100A8+ tumor cells (TC) and S100A8+ immune cells (IC). S100A8+ TCs and S100A8+ ICs show a weak positive correlation (rho = 0.260) in pre-invasive carcinoma (**a**) and moderate positive correlation (rho = 0.452) in invasive carcinoma (**b**)

S100A8 was expressed in both TCs and ICs of pre-invasive and invasive carcinomas (Fig. 1). In pre-invasive carcinoma, S100A8 expression was detected in up to 90% of TCs, and S100A8+ ICs were found in up to 50% of a tumor area. In invasive carcinoma, S100A8+ TCs comprised up to 100% of TCs, and S100A8+ ICs were also found in up to 50% of a tumor area. S100A8 expression in TCs and ICs showed a weak positive correlation in pre-invasive carcinoma (rho = 0.260) and a moderate positive correlation in invasive carcinoma (rho = 0.452) as shown in the scatter plots (Fig. 2).

When comparing the infiltration of S100A8+ ICs between pre-invasive carcinoma and invasive carcinoma as a continuous variable (Table 1), S100A8+ ICs were significantly higher in invasive carcinomas than in pre-invasive carcinomas (*p* = 0.010). In subgroup analysis by HR status, the difference was also apparent in HR-negative subgroup (*p* = 0.014) but not in HR-positive subgroup (*p* = 0.872). The proportion of S100A8+ TCs did not differ between pre-invasive and invasive carcinomas in the whole group (*p* = 0.544) and in the HR-positive subgroup (*p* = 0.228); however, it tended to be higher in invasive carcinomas than in pre-invasive carcinomas in the HR-negative subgroup (*p* = 0.081).

**Table 1:** Comparison of S100A8 expression between pre-invasive carcinoma and invasive carcinoma

	Pre-invasive carcinoma	Invasive carcinoma	*p* value
Total			
S100A8+ TC (%)	0.00 (0.00–5.00)	0.00 (0.00–2.63)	0.544
S100A8+ IC (%)	3.00 (1.00–5.00)	4.33 (2.33–8.33)	0.010
HR+ subgroup			
S100A8+ TC (%)	0.00 (0.00–0.00)	0.00 (0.00–0.00)	0.228
S100A8+ IC (%)	3.00 (1.00–5.00)	3.33 (1.67–5.00)	0.872
HR- subgroup			
S100A8+ TC (%)	10.00 (8.75–36.67)	5.00 (0.00–40.00)	0.081
S100A8+ IC (%)	5.00 (2.00–9.17)	7.67 (4.33–15.00)	0.014

*P* values are calculated by Mann–Whitney test. Data are presented as median value (interquartile range)

*TC* tumor cell, *IC* immune cell, *HR* hormone receptor

### *S100A8*+ *tumor cells and immune cells in relation to clinicopathologic features of tumor*

Relationships between the presence of S100A8+ TCs or ICs and various clinicopathologic features of the tumors are summarized in Supplementary Tables S3 and S4. In pre-invasive carcinoma, the presence of S100A8+ TCs was associated with aggressive clinicopathologic features of tumor including a large extent of tumor, high nuclear grade, comedo-type necrosis, ER negativity, PR negativity, positive HER2 status, high Ki-67 index, and p53 overexpression (all *p* < 0.05). Infiltration of S100A8+ ICs showed an association with only ER negativity (*p* = 0.030) and tended to be associated with comedo-type necrosis (*p* = 0.081) and high Ki-67 index (*p* = 0.087). In invasive carcinoma, the presence of S100A8+ TCs and ICs was commonly associated with high histologic grade, ER negativity, PR negativity, HER2 positivity, and p53 overexpression (all *p* < 0.001).

### *Impact of S100A8*+ *tumor cells and immune cells on clinical outcome of the patients*

**Fig. 3:**
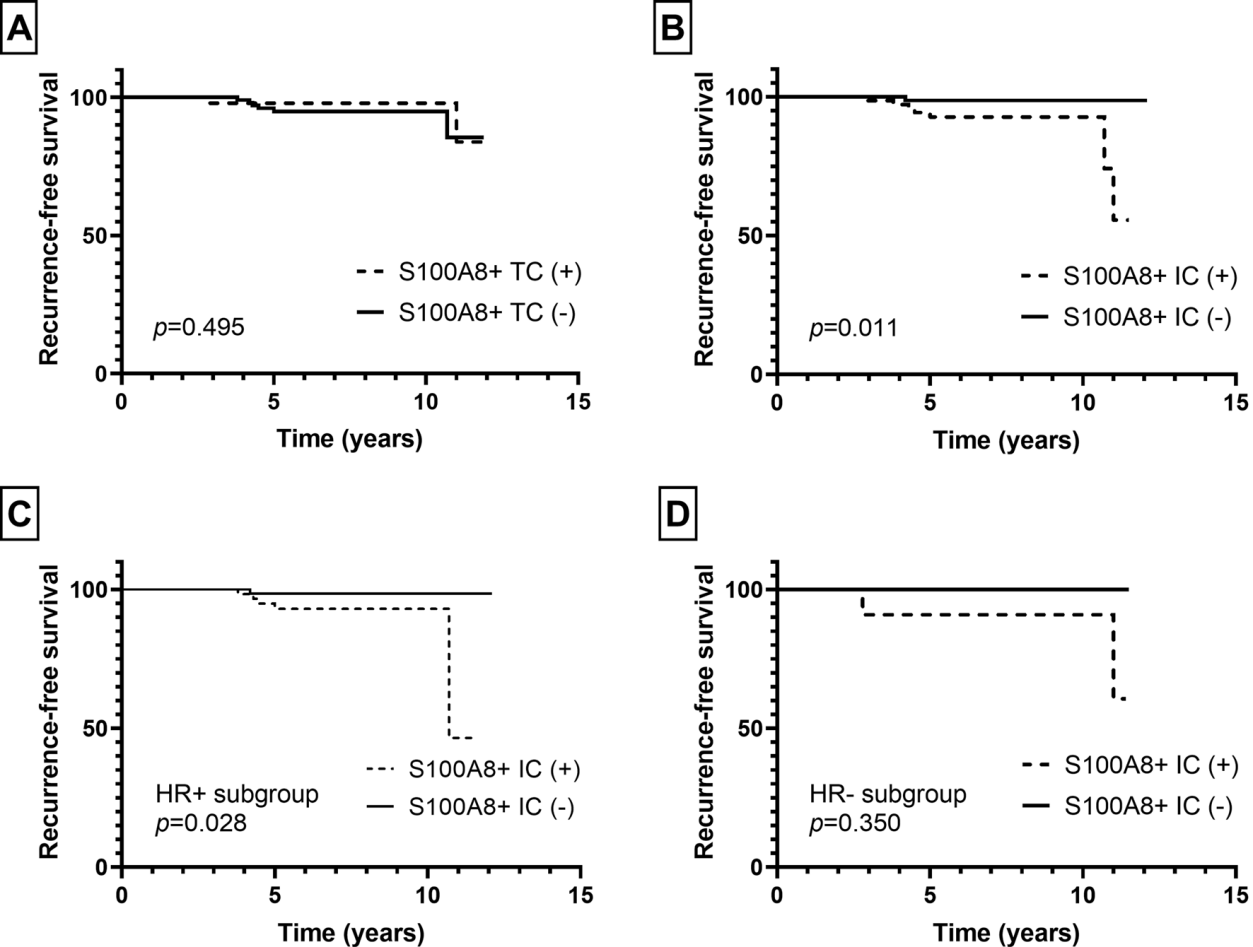
Kaplan–Meier survival curves according to S100A8+ tumor cell (TC) and S100A8+ immune cell (IC) in pre-invasive carcinoma. While S100A8+ TC (**a**) is not associated with ipsilateral breast recurrence, infiltration of S100A8+ IC (**b**) is associated with decreased recurrence-free survival in pre-invasive carcinoma. In subgroup analyses, infiltration of S100A8+ IC is associated with poor recurrence-free survival in the hormone receptor (HR)-positive subgroup (**c**), but not in the HR-negative subgroup (**d**)

Next, we evaluated the patients’ clinical outcome in relation to the presence of S100A8+ TCs or infiltration of S100A8+ ICs in pre-invasive and invasive carcinomas. As for patients with pre-invasive carcinoma, the mean follow-up period was 6.7 years (standard deviation, 3.0 years) during which 8 patients developed ipsilateral breast recurrence. In survival analyses, infiltration of S100A8+ ICs, but not the presence of S100A+ TCs, was associated with ipsilateral breast recurrence (*p* = 0.011, *p* = 0.495, respectively; Fig. 3). In subgroup analyses according to HR status, infiltration of S100A8+ ICs was associated with ipsilateral breast recurrence in the HR-positive subgroup, but not in the HR-negative subgroup (*p* = 0.028, *p* = 0.350, respectively; Fig. 3).

**Fig. 4:**
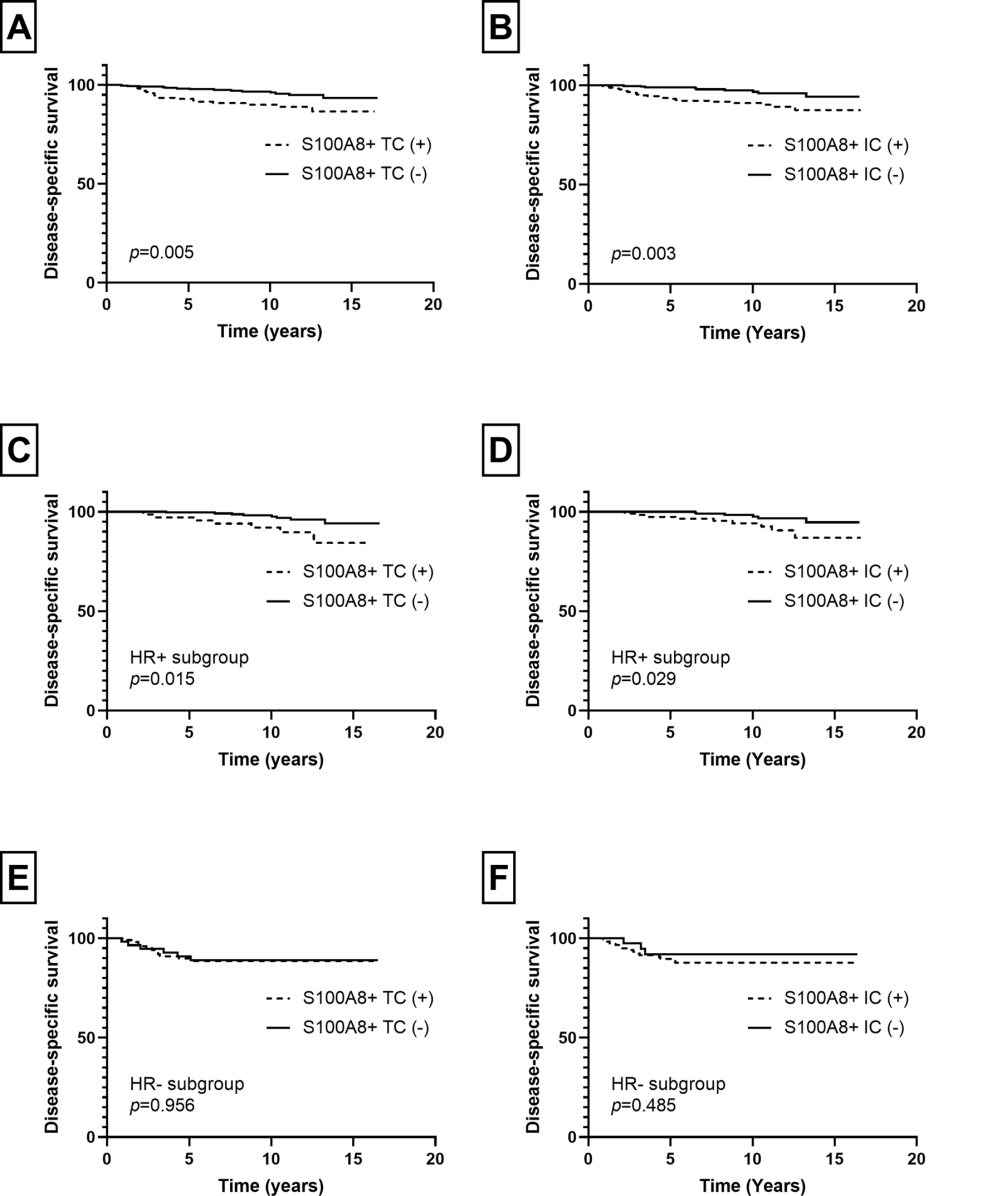
Kaplan–Meier survival curves according to infiltration of S100A8+ tumor cell (TC) and S100A8+ immune cell (IC) in invasive breast carcinoma. As a whole, presence of S100A8+ TC (**a**) and infiltration of S100A8+ IC (**b**) are associated with poor disease-specific survival. In hormone receptor (HR)-positive subgroup, S100A8+ TC (**c**) and S100A8+ IC (**d**) are also associated with decreased disease-specific survival. In HR-negative subgroup, there is no statistical difference in survival in relation to S100A8+ TC (**e**) or S100A8+ IC (**f**) infiltration

In patients with invasive carcinoma, the mean follow-up period was 9.4 years (standard deviation, 4.0 years) during which 32 patients revealed cancer-related death. In survival analyses, the presence of S100A8+ TCs and infiltration of S100A8+ ICs were associated with poor disease-specific survival (*p* = 0.005, *p* = 0.003, respectively; Fig. 4). The same difference in survival was also observed in HR-positive subgroup (S100A8+ TCs, *p* = 0.015; S100A8+ ICs, *p* = 0.029; Fig. 4). However, in HR-negative subgroup, there was no statistical difference in disease-specific survival in relation to S100A8+ TCs or ICs (*p* = 0.956, *p* = 0.485, respectively; Fig. 4). In multivariate analyses (Table 2), infiltration of S100A8+ ICs (*p* = 0.041) was revealed as an independent poor prognostic factor along with nodal metastasis, lymphovascular invasion, and hormone receptor negativity (*p* = 0.019, *p* = 0.048, *p* = 0.013, respectively). However, S100A8+ TC was not proven an independent prognostic factor (*p* = 0.237).

**Table 2:** Univariate and multivariate analyses of disease-specific survival in invasive carcinoma

Variable	Category	Univariate analysis		Multivariate analysis	
		Hazard ratio (95% CI)	*p* value	Hazard ratio (95% CI)	*p* value
Age	< 50 years vs. ≥ 50 years	0.818 (0.407–1.664)	0.572	–	–
T stage	T1 vs. T2–4	2.193 (1.014–4.743)	0.046	1.377 (0.615–3.083)	0.437
N stage	N0 vs. N1–N3	3.187 (1.474–6.895)	0.003	2.761 (1.184–6.437)	0.019
Histologic grade	I and II vs. III	2.644 (1.223–5.716)	0.013	1.527 (0.612–3.811)	0.364
LVI	Absent vs. present	3.280 (1.517–7.091)	0.003	2.342 (1.008–5.444)	0.048
Hormone receptor	Negative vs. positive	0.369 (0.184–0.738)	0.005	0.390 (0.186–0.820)	0.013
HER2	Negative vs. positive	1.158 (0.520–2.579)	0.719	–	–
Ki-67 index	Low vs. high	2.058 (1.016–4.167)	0.045	0.822 (0.324–2.086)	0.680
S100A8+ TCs	Negative vs. positive	2.594 (1.290–5.215)	0.007	1.594 (0.736–3.452)	0.237
S100A8+ ICs	Negative vs. positive	3.017 (1.396–6.522)	0.005	2.345 (1.034–5.316)	0.041

*CI* confidence interval, *LVI* lymphovascular invasion, *TC* tumor cell, *IC* immune cell

**Fig. 5:**
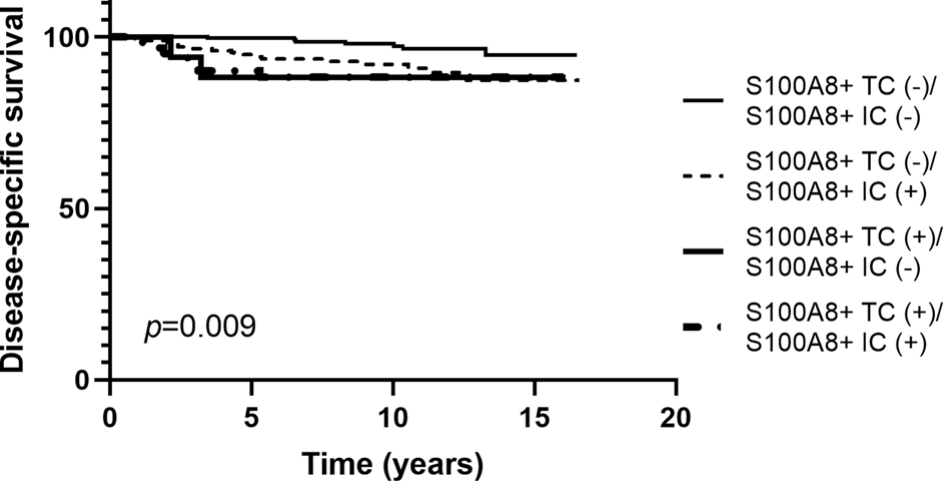
Kaplan–Meier survival curves according to combined analyses of S100A8+ tumor cell (TC) and S100A8+ immune cell (IC) infiltration in invasive carcinoma. Among the four combined groups, only the S100A8+ TC (−)/S100A8+ IC (−) group shows better disease-specific survival compared to the other groups

As S100A8 expression in TCs and ICs was not always concordant, survival analyses were performed using the combination of S100A8+ TCs and ICs. Disease-specific survival was different among the four combined groups (*p* = 0.009; Fig. 5) with the best clinical outcome belonging to S100A8+ TC (−)/S100A8+ IC (−) group. However, there was no difference in survival among S100A8+ TC (+)/S100A8+ IC (−) group, S100A8 + TC (−)/S100A8 + IC ( +) group, and S100A8+ TC (+)/S100A8+ IC (+) group.

### *Association of S100A8*+ *immune cells with other immune cell subset infiltration*

As MDSCs are known to be associated with regulatory T cell infiltration and PD-L1 induction, we analyzed the correlation between S100A8+ IC, TIL subsets, and PD-L1+ IC infiltration in pre-invasive and invasive carcinomas. In pre-invasive carcinoma, infiltration of S100A8+ IC revealed weak positive correlations with infiltration of CD4+, CD8+, and FOXP3+ TIL and PD-L1+ IC (rho 0.209–0.281; Supplementary Table S5). In invasive carcinoma, infiltration of S100A8+ IC showed a weak positive correlation with infiltration of CD4+ TIL (rho = 0.263) and a moderate positive correlation with infiltration of CD8+ and FOXP3+ TIL and PD-L1+ IC (rho = 0.474, 0.482 and 0.525, respectively, Supplementary Table S3). Table 3 shows the distribution of CD4+, CD8+, and FOXP3+ TILs and the frequency of PD-L1+ IC in relation to S100A8+ IC. Generally, TIL subset infiltration was significantly higher in S100A8+ IC (+) group than in S100A8+ IC (−) group in both pre-invasive and invasive carcinoma as a whole (all *p* < 0.01). PD-L1 + IC was more frequently observed in S100A8+ IC (+) group compared to S100A8+ IC (−) group in both pre-invasive and invasive carcinomas (*p* = 0.006, *p* < 0.001, respectively). In subgroup analyses by HR status, HR-positive subgroup showed a similar pattern as the whole group in both pre-invasive and invasive carcinomas. In HR-negative subgroup, pre-invasive carcinoma did not show a difference in TIL subset and PD-L1+ IC infiltration in relation to S100A8+ IC, whereas invasive carcinoma revealed significant higher TIL and PD-L1+ IC infiltration in S100A8+ IC (+) group compared to S100A8+ IC (−) group.

**Table 3:** Comparison of immune cell subset infiltration in relation to S100A8 + immune cells

Immune cell subset	Pre-invasive carcinoma^a^		*p* value	Invasive carcinoma^b^		*p* value
	S100A8+ IC (−)	S100A8+ IC (+)		S100A8+ IC (−)	S100A8+ IC (+)	
Total						
CD4+ TIL	15.33 (2.33–34.00)	35.00 (9.67–90.17)	< 0.001	76.00 (30.50–136.50)	127.50 (52.50–195.50)	< 0.001
CD8+ TIL	9.0 (4.33–19.33)	15.00 (8.83–41.33)	< 0.001	59.00 (29.50–110.00)	166.00 (81.50–290.50)	< 0.001
FOXP3+ TIL	0.00 (0.00–0.00)	0.00 (0.00–4.00)	0.006	5.00 (2.00–11.00)	17.00 (9.00–28.00)	< 0.001
PD-L1+ IC	9/92 (9.8)	21/82 (25.6)	0.006	38/161 (23.6)	100/146 (68.5)	< 0.001
HR+ subgroup						
CD4+ TIL	14.33 (2.00–29.33)	43.00 (9.33–91.33)	< 0.001	76.50 (34.25–136.75)	109.50 (46.50–188.00)	0.023
CD8+ TIL	9.00 (4.33–17.83)	14.67 (9.00–39.00)	< 0.001	56.00 (29.00–99.50)	133.00 (54.50–277.75)	< 0.001
FOXP3+ TIL	0.00 (0.00–0.00)	0.00 (0.00–4.00)	0.012	5.00 (2.00–10.00)	15.00 (7.00–20.00)	< 0.001
PD-L1+ IC	8/85 (9.4)	15/68 (22.1)	0.030	22/132 (16.7)	45/80 (56.3)	< 0.001
HR- subgroup						
CD4+ TIL	53.3 (39.17–81.08)	27.67 (18.08–84.33)	0.689	71.00 (15.00–131.50)	138.50 (63.00–203.25)	0.012
CD8+ TIL	18.50 (6.08–38.92)	19.00 (6.58–65.50)	0.585	77.00 (35.00–163.00)	195.00 (108.50–306.50)	< 0.001
FOXP3+ TIL	0.50 (0.00–5.92)	1.00 (0.00–5.75)	0.904	8.00 (3.00–11.50)	19.00 (12.00–33.25)	< 0.001
PD-L1+ IC	1/7 (14.3)	6/14 (42.9)	0.337	16/29 (55.2)	55/66 (83.3)	0.004

*P* values are calculated by Mann–Whitney *U* test or Chi-square test. Data are presented as median (interquartile range) for TIL and frequency (%) for PD-L1+ IC

*TIL* tumor-infiltrating lymphocyte, *IC* immune cell, *HR* hormone receptor

^a^In pre-invasive carcinomas, data on immune cell subset infiltration were missing in six cases: CD4+ TIL in one case, FOXP3+ TI in two cases, CD8+ TIL in one case, PD-L1+ IC in two cases

^b^In invasive carcinomas, data on immune cell subset infiltration were missing in three cases: CD8+ TIL in one case, FOX3+ TIL in one case, and PD-L1+ IC in one case

### *Combined effect of S100A8*+ *immune cells and other immune cell subset infiltration on clinical outcome*

**Fig. 6:**
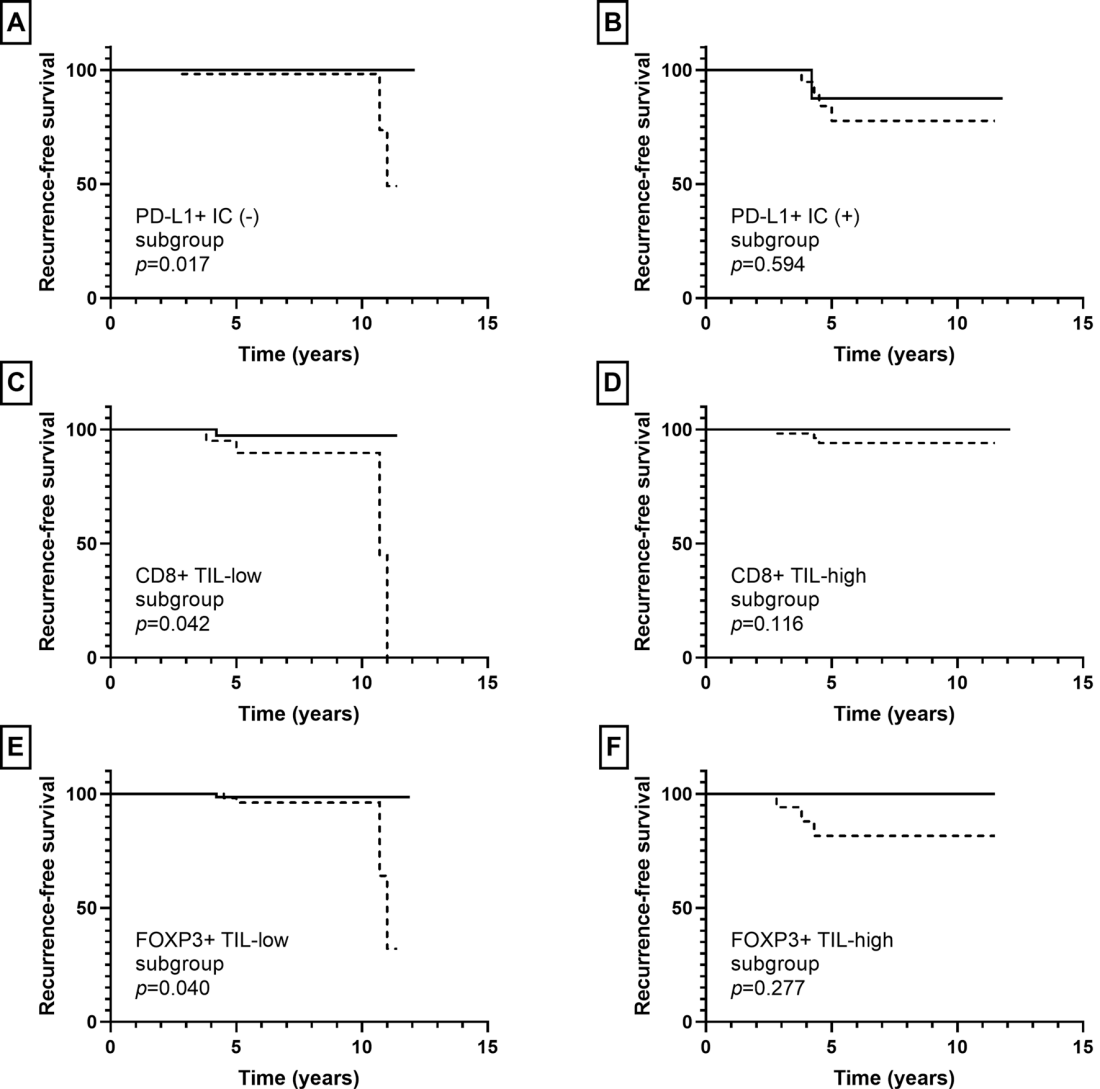
Kaplan–Meier survival curves according to combination of S100A8+ immune cell (IC) and other IC subset infiltration in pre-invasive carcinoma. Solid line indicates S100A8+ IC (−) group and dotted line indicates S100A8+ IC (+) group. S100A8+ IC (+) group is associated with shorter ipsilateral breast recurrence-free survival compared to S100A8+ IC (−) group in the PD-L1+ IC (−) (**a**), CD8+ TIL-low (**c**), and FOXP3+ TIL-low (**e**) subgroups. No statistical difference in recurrence-free survival is observed between S100A8+ IC (+) and S100A8+ IC (−) groups in the PD-L1+ IC (+) (**b**), CD8+ TIL-high (**d**), and FOXP3+ TIL-high (**f**) subgroups

Besides S100A8+ IC, the presence of PD-L1+ IC was associated with ipsilateral breast recurrence in pre-invasive carcinoma (*p* = 0.001). Low infiltration of CD8+ TIL and high infiltration of FOX3+ TIL tended to be associated with ipsilateral breast recurrence (*p* = 0.111, *p* = 0.103, respectively); while CD4+ TIL infiltration did not show an association with ipsilateral breast recurrence (*p* = 0.421). In subgroup analyses, infiltration of S100A8+ ICs was associated with ipsilateral breast recurrence in the PD-L1+ IC (−), CD8+ TIL-low, and FOXP3+ TIL-low subgroups (*p* = 0.017, *p* = 0.042, *p* = 0.040, respectively), but not in the PD-L1+ IC (+), CD8+ TIL-high, and FOXP3+ TIL-high subgroups (*p* = 0.594, *p* = 0.116, *p* = 0.277, respectively) (Fig. 6).

**Fig. 7:**
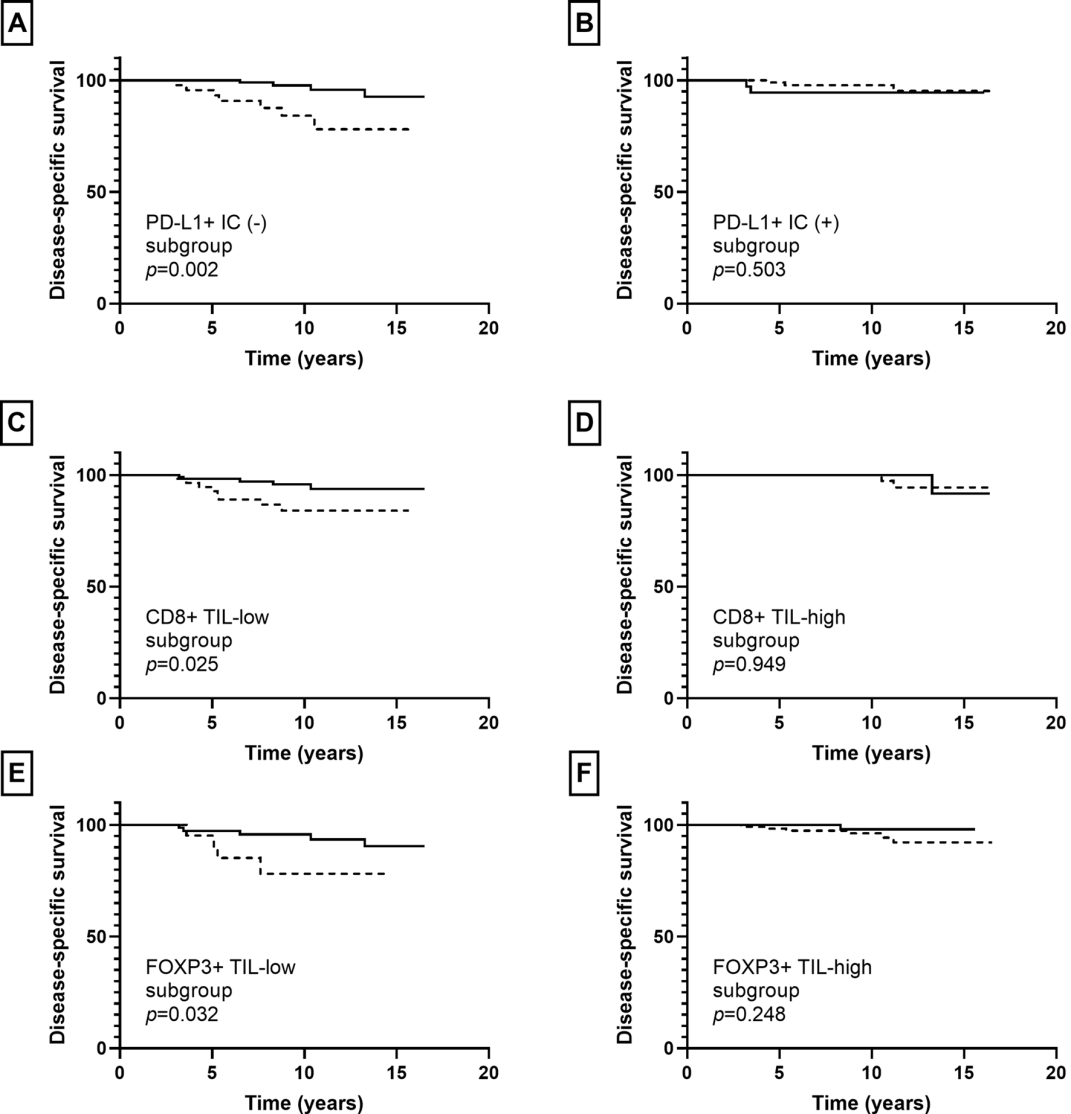
Kaplan–Meier survival curves according to combination of S100A8+ immune cell (IC) and other IC subset infiltration in invasive carcinoma. Solid line indicates S100A8+ IC (−) group and dotted line indicates S100A8+ IC (+) group. S100A8+ IC (+) group shows poor disease-specific survival in the PD-L1+ IC (−) (**a**), CD8+ TIL-low (**c**), and FOXP3+ TIL-low (**e**) subgroups. No survival differences are found between S100A8+ IC (+) and S100A8+ IC (−) groups in the PD-L1+ IC (+) (**b**), CD8+ TIL-high (**d**), and FOXP3 + TIL-high (**f**) subgroups

In invasive carcinoma, infiltration of CD4+, CD8+, and FOXP3+ TIL showed an association with disease-specific survival (*p* = 0.015, *p* = 0.039, *p* = 0.069, respectively) albeit borderline significance for FOX3P3+ TIL. The presence of PD-L1+ IC was not associated with patients’ disease-specific survival (*p* = 0.213). In subgroup analyses, infiltration of S100A8+ ICs was associated with decreased disease-specific survival in the PD-L1+ IC (−), CD8+ TIL-low, and FOXP3+ TIL-low subgroups (*p* = 0.002, *p* = 0.025, *p* = 0.032, respectively), but not in the PD-L1+ IC (+), CD8 + TIL-high, and FOXP3+ TIL-high subgroups (*p* = 0.503, *p* = 0.949, *p* = 0.248, respectively) (Fig. 7).

## Discussion

In this study, we compared S100A8 expression in pre-invasive and invasive carcinoma of the breast and observed significantly higher infiltration of S100A8+ ICs in invasive carcinoma than pre-invasive carcinoma. Many studies on circulating MDSCs have reported that MDSCs accumulate systemically in the body from the pre-invasive stage and exerts the same immunosuppressive role as they do in various cancers including the breast [20, 21, 29, 30]. Particularly, Clark et al. [20] revealed that circulating MDSCs progressively accumulate from normal to pre-invasive to invasive pancreatic cancer. Put together, we can deduce that MDSCs already exist in a pre-invasive tumor and increase in number as the tumor progresses. Additionally, we found that the difference in S100A8+ IC infiltration between pre-invasive and invasive carcinomas was evident in HR-negative tumors, but not in HR-positive tumors. However, as the number of HR-negative pre-invasive carcinoma was quite small (*n* = 21), and HR-positive tumors generally showed no S100A8+ IC infiltration, further large-series studies are warranted to confirm this finding.

In contrast to S100A8+ IC infiltration, the presence of S100A8 + TCs did not differ between pre-invasive and invasive carcinomas. Previously, Arai et al. [15] reported that S100A8 expression was found in 66.7% (16/24) of ductal carcinoma in situ (DCIS) and 45.5% (46/101) of invasive ductal carcinoma with a slightly higher frequency in DCIS. In our study, in terms of frequency of positive cases, S100A8+ TCs was found in 30.7% (54/176) of pre-invasive carcinomas and 33.4% (175/524) of invasive carcinomas without a statistical difference (data not shown). Similarly, Choi et al. [19] have reported no difference in S100A8 expression in TCs between primary invasive ductal carcinoma and adjacent DCIS. In addition, we have shown that S100A8 expression in TCs was associated with poor clinicopathologic features in both pre-invasive and invasive carcinomas as reported in previous studies [15–19]. Thus, it seems that S100A8 is expressed in TCs of pre-invasive carcinoma just as much as invasive carcinoma in association with aggressive clinicopathologic features of tumor.

In survival analyses, infiltration of S100A8+ ICs was associated with ipsilateral breast recurrence in pre-invasive carcinoma, and it was found to be an independent poor prognostic factor in invasive carcinoma. These results suggest that S100A8+ ICs play an important role during progression of both pre-invasive and invasive carcinomas. Thus, pre-invasive and invasive breast carcinomas with high S100A8+ IC infiltration could be a target for close observation and aggressive additional treatment. Additionally, in this study, decreased survival was evident in HR-positive subgroup, but not in HR-negative subgroup. Miller et al. [17] demonstrated a clear difference in overall survival in both HR-positive and -negative subgroups using automated quantitative immunofluorescence. This discrepancy in results seems to be from the difference in evaluation method since they used different cutoffs for HR-positive and -negative tumors. Even though we tried using different cutoffs in HR-negative tumors, we obtained similar results (data not shown). Nonetheless, our finding that prognostic significance of S100A8+ ICs depends on HR status gives an important clue to the role of S100A8+ ICs in breast cancer progression since HR-positive tumors are generally less immunogenic compared to HR-positive tumors.

Furthermore, in combined analyses of S100A8+ IC and other IC subset infiltration, we found that infiltration of S100A8+ IC was associated with decreased disease-specific survival in the PD-L1+ IC (−), CD8+ TIL-low, and FOXP3+ TIL-low subgroups in invasive carcinomas. Similarly, infiltration of S100A8+ IC was associated with decreased ipsilateral breast recurrence-free survival in the same subgroups in pre-invasive carcinomas. That is, the prognostic significance of S100A8+ ICs was more prominent in less immunogenic tumors. Thus, it can be postulated that MDSCs play a role in tumor progression mainly via a non-immune mechanism, such as neovascularization and promotion of invasion and metastasis rather than via anti-tumor immunity. In line with our results, Drews-Elger et al. [14] demonstrated that recruitment of S100A8+ myeloid cells in xenograft models of breast cancer enhance tumor progression independent of their suppressive activity on T cells using immunosuppressed mouse models. In this study, we have shown for the first time the positive relationship between S100A8+ ICs and various IC subsets and their combined prognostic impact using human breast cancer tissues. Further investigations are needed to elucidate our findings.

S100A8+ ICs showed a weak to moderate positive correlation with other IC subset infiltration, and CD4+, CD8+, FOXP3+ TIL and PD-L1+ IC infiltration was significantly higher in S100A8+ IC (+) group compared to S100A8+ IC (−) group. The positive correlation between S100A8+ ICs and other IC subset was more prominent in invasive carcinoma than in pre-invasive carcinoma. Increased FOXP3+ TIL infiltration in S100A8+ IC (+) group is consistent with the fact that MDSCs induce regulatory T cells [31–33]. In this study, CD8+ TIL infiltration also showed a positive correlation with S100A8+ IC infiltration in pre-invasive and invasive carcinomas. However, the anti-tumor activity of CD8+ TILs in S100A8+ ICs (+) group remains unclear since interference by MDSCs can result in CD8+ T cell tolerance [34–36]. Positive correlation between PD-L1+ ICs and S100A8+ ICs can also be explained by the finding that tumor-infiltrating MDSCs show upregulated expression of PD-L1 [37]. It seems that the amount and composition of TIL subsets go through a dynamic change from pre-invasive carcinoma to invasive carcinoma, and this change is likely to be related to the early-existing S100A8+ ICs.

There are several limitations in this study. First, S100A8 expression was read manually and TMAs were used instead of evaluating whole sections though some countermeasures were adopted to minimize the limitation: setting strict reading criteria, categorization of continuous values, and use of multiple TMA cores. Second, caution must be exercised when interpreting of S100A8+ ICs as MDSCs since S100A8 is only a surrogate marker of MDSCs and other immune cells such as macrophages and neutrophils can also express S100A8. MDSC can be classified into polymorphonuclear MDSC and monocytic MDSC, each having a different role in a tumor. For detailed phenotypic analysis, follow-up studies using other objective methods such as flow cytometry or multiplex immunohistochemistry is needed to elucidate the role of specific subset of MDSCs.

In conclusion, infiltration of S100A8+ IC was associated with aggressive clinicopathological features and poor clinical outcome in our breast cancer patients. S100A8+ ICs were already present in early stage of breast cancer and were associated with increased CD4+, CD8+, FOXP3+ TILs and PD-L + ICs infiltration. In combined survival analyses, prognostic impact of S100A8+ ICs was stronger in HR-positive, PD-L1+ IC (−), CD8+ TIL-low and FOXP3+ TIL-low subgroups, which are characterized as less immunogenic tumors. In those breast cancer patients, evaluation of S100A8+ IC may be helpful in planning additional treatment. Further investigation on MDSCs and therapeutic intervention targeting MDSC-associated molecules including S100A8 is warranted.

## Electronic supplementary material

Below is the link to the electronic supplementary material.

Supplementary file1 (PDF 269 KB)

## Data Availability

The datasets generated during and/or analyzed during the current study are available from the corresponding author on reasonable request.
