# Supplementary material for: Prognostic significance of S100A8-positive immune cells in relation to other immune cell infiltration in pre-invasive and invasive breast cancers
Source: Cancer Immunol Immunother. 2020 Nov 4;70(5):1365–78. doi: 10.1007/s00262-020-02776-5 (PMC8053168; doi:10.1007/s00262-020-02776-5)
Supplement: Supplementary file 1 — Supplementary file1 (PDF 269 KB) [file 262_2020_2776_MOESM1_ESM.pdf]

**Supplementary Table S1. Clinicopathologic characteristics of pre-invasive carcinomas**

| **Characteristic** | **No. (n=176)** |
|---|---|
| Age at diagnosis, years | |
| Mean ± standard deviation | 48.3 ± 10.0 |
| Follow-up, years | |
| Mean ± standard deviation | 6.7 ± 3.0 |
| Extent, cm | |
| Mean ± standard deviation | 3.2 ± 2.1 |
| Histological subtype | |
| DCIS | 175 (99.4) |
| Mixed DCIS and LCIS | 1 (0.6) |
| Nuclear grade | |
| Low | 11 (6.3) |
| Intermediate | 98 (55.7) |
| High | 67 (38.1) |
| Comedo-type necrosis | |
| Absent | 133 (75.6) |
| Present | 43 (24.4) |
| Estrogen receptor | |
| Positive | 154 (87.5) |
| Negative | 22 (12.5) |
| Progesterone receptor | |
| Positive | 134 (76.1) |
| Negative | 83 (23.9) |
| HER2 status | |
| Negative | 151 (85.8) |
| Positive | 25 (14.2) |
| Ki67 index | |
| Low (<10%) | 165 (93.8) |
| High (≥10%) | 11 (6.3) |
| P53 overexpression | |
| Absent | 155 (88.1) |
| Present | 21 (11.9) |
| Subtype | |
| Luminal A | 127 (72.2) |
| Luminal B | 28 (15.9) |
| HER2+ | 8 (4.5) |
| Triple negative | 13 (7.4) |
| Adjuvant radiation therapy | |
| Not received | 71 (40.3) |
| Received | 105 (59.7) |
| Adjuvant hormonal therapy | |
| Not received | 105 (59.7) |
| Received | 71 (40.3) |

Unless specified, number in parenthesis indicates percentage.

DCIS, ductal carcinoma in situ; LCIS, lobular carcinoma in situ

**Supplementary Table S2. Clinicopathologic characteristics of invasive carcinomas**

| **Characteristic** | **No. (n=524)** |
|---|---|
| Age at diagnosis, years | |
| Mean ± standard deviation | 51.2 ± 11.8 |
| Follow up, years | |
| Mean ± standard deviation | 9.4 ± 4.0 |
| T stage | |
| T1 | 246 (47.0) |
| T2 | 251 (47.9) |
| T3 | 19 (3.6) |
| T4 | 8 (1.5) |
| N stage | |
| N0 | 300 (57.3) |
| N1 | 137 (26.1) |
| N2 | 50 (9.5) |
| N3 | 37 (7.1) |
| Histologic subtype | |
| Invasive carcinoma of no special type | 456 (87.0) |
| Invasive lobular carcinoma | 20 (3.8) |
| Mucinous carcinoma | 15 (2.9) |
| Metaplastic carcinoma | 14 (2.7) |
| Others | 19 (3.6) |
| Histologic grade | |
| Low | 94 (17.9) |
| Intermediate | 166 (31.7) |
| High | 264 (50.4) |
| Lymphovascular invasion | |
| Absent | 296 (56.5) |
| Present | 228 (43.5) |
| Estrogen receptor | |
| Positive | 353 (67.4) |
| Negative | 171 (32.6) |
| Progesterone receptor | |
| Positive | 278 (53.1) |
| Negative | 246 (46.9) |
| HER2 status | |
| Negative | 404 (77.1) |
| Positive | 120 (22.9) |
| Ki67 index | |
| Low (<20%) | 301 (57.4) |
| High (≥20%) | 223 (42.6) |
| P53 overexpression | |
| Absent | 386 (73.7) |
| Present | 138 (26.3) |
| Subtype | |
| Luminal A | 225 (42.9) |
| Luminal B | 139 (26.5) |
| HER2+ | 64 (12.2) |
| Triple negative | 96 (18.3) |
| Neo-adjuvant chemotherapy | |
| Not received | 491 (93.7) |
| Received | 33 (6.3) |
| Adjuvant chemotherapy* | |
| Not received | 87 (16.6) |
| Received | 431 (85.3) |
| Adjuvant HER2-targeted therapy* | |
| Not received | 460 (87.8) |
| Received | 58 (11.1) |
| Adjuvant radiation therapy* | |
| Not received | 204 (38.9) |
| Received | 314 (59.9) |
| Adjuvant hormonal therapy* | |
| Not received | 159 (30.3) |
| Received | 359 (68.5) |

Unless specified, number in parenthesis indicate percentage.

*Information on adjuvant therapy could not be assessed in 6 cases due to follow-up loss.

**Supplementary Table S3. Relationship between S100A8 expression in tumor cells and immune cells and clinicopathological features of pre-invasive carcinoma**

| **Clinicopathological feature** | **S100A8+ TC** | | ***p* value** | **S100A8+ IC** | | ***p* value** |
|---|---|---|---|---|---|---|
| | **Negative** | **Positive** | | **Negative** | **Positive** | |
| Extent | | | 0.012 | | | 0.238 |
| <3.2cm | 61 (50.0) | 38 (70.4) | | 49 (52.1) | 50 (61.0) | |
| ≥3.2cm | 61 (50.0) | 16 (29.6) | | 45 (47.9) | 32 (39.0) | |
| Nuclear grade | | | 0.004 | | | 0.594 |
| Low | 10 (8.2) | 1 (1.9) | | 7 (7.4) | 4 (4.9) | |
| Intermediate | 75 (61.5) | 23 (42.6) | | 54 (57.4) | 44 (53.7) | |
| High | 37 (30.3) | 30 (55.6) | | 33 (35.1) | 34 (41.5) | |
| Comedo-type necrosis | | | 0.027 | | | 0.081 |
| Absent | 98 (80.3) | 35 (64.8) | | 76 (80.9) | 57 (69.5) | |
| Present | 24 (19.7) | 19 (35.2) | | 18 (19.1) | 25 (30.5) | |
| ER | | | <0.001 | | | 0.030 |
| Positive | 119 (97.5) | 35 (64.8) | | 87 (92.6) | 67 (81.7) | |
| Negative | 3 (2.5) | 19 (35.2) | | 7 (7.4) | 15 (18.7) | |
| PR | | | <0.001 | | | 0.389 |
| Positive | 110 (90.2) | 24 (44.4) | | 74 (78.7) | 60 (73.2) | |
| Negative | 12 (9.8) | 30 (55.6) | | 20 (21.3) | 22 (26.8) | |
| HER2 status | | | 0.001 | | | 0.147 |
| Negative | 112 (91.8) | 39 (72.2) | | 84 (89.4) | 67 (81.7) | |
| Positive | 10 (8.2) | 15 (27.8) | | 10 (10.6) | 15 (18.3) | |
| Ki67 index | | | 0.036 | | | 0.087 |
| Low (<10%) | 111 (91.0) | 43 (79.6) | | 86 (91.5) | 68 (82.9) | |
| High (≥10%) | 11 (9.0) | 11 (20.4) | | 8 (8.5) | 14 (17.1) | |
| P53 overexpression | | | 0.005 | | | 0.134 |
| Absent | 113 (92.6) | 42 (77.8) | | 86 (91.5) | 69 (84.1) | |
| Present | 9 (7.4) | 12 (22.2) | | 8 (8.5) | 13 (15.9) | |
| Subtype | | | <0.001 | | | 0.215 |
| Luminal A | 101 (82.8) | 26 (48.1) | | 73 (77.7) | 54 (65.9) | |
| Luminal B | 18 (14.8) | 10 (18.5) | | 14 (14.9) | 14 (17.1) | |
| HER2+ | 1 (0.8) | 7 (13.0) | | 3 (3.2) | 5 (6.1) | |
| Triple negative | 2 (1.6) | 11 (20.4) | | 4 (4.3) | 9 (11.0) | |

*P* values are calculated by Chi-square or Fisher's exact test. Number in parenthesis indicates percentage.

TC, tumor cell; IC, immune cell; ER, estrogen receptor; PR, progesterone receptor

**Supplementary Table S4. Relationship between S100A8 expression in tumor cells and immune cells and clinicopathological features of invasive carcinoma**

| Clinicopatholgical features | S100A8+ TC | | *p* value | S100A8+ IC | | *p* value |
|---|---|---|---|---|---|---|
| | Negative | Positive | | Negative | Positive | |
| T stage | | | 0.689 | | | 0.064 |
| T1 | 166 (47.6) | 80 (45.7) | | 142 (50.7) | 104 (42.6) | |
| T2-T4 | 183 (52.4) | 95 (54.3) | | 138 (49.3) | 140 (57.4) | |
| N stage | | | 0.880 | | | 0.068 |
| N0 | 199 (57.0) | 101 (57.7) | | 150 (53.6) | 150 (61.5) | |
| N1-N3 | 150 (43.0) | 74 (42.3) | | 130 (46.4) | 94 (38.5) | |
| Histologic grade | | | <0.001 | | | <0.001 |
| Low | 85 (24.4) | 9 (5.1) | | 72 (25.7) | 22 (9.0) | |
| Intermediate | 136 (39.0) | 30 (17.1) | | 124 (44.3) | 42 (17.2) | |
| High | 128 (36.7) | 136 (77.7) | | 84 (30.0) | 180 (73.8) | |
| LVI | | | 0.557 | | | 0.393 |
| Absent | 194 (55.6) | 102 (58.3) | | 163 (58.2) | 133 (54.5) | |
| Present | 155 (44.4) | 73 (41.7) | | 117 (41.8) | 111 (45.5) | |
| ER | | | <0.001 | | | <0.001 |
| Positive | 287 (82.2) | 66 (37.7) | | 236 (84.3) | 117 (48.0) | |
| Negative | 62 (17.8) | 109 (62.3) | | 44 (15.7) | 127 (52.0) | |
| PR | | | <0.001 | | | <0.001 |
| Positive | 227 (65.0) | 51 (29.1) | | 187 (66.8) | 91 (37.3) | |
| Negative | 122 (35.0) | 124 (70.9) | | 93 (33.2) | 153 (62.7) | |
| HER2 status | | | <0.001 | | | <0.001 |
| Negative | 56 (16.0) | 64 (36.6) | | 42 (15.0) | 78 (32.0) | |
| Positive | 293 (84.0) | 111 (63.4) | | 238 (85.0) | 166 (68.0) | |
| Ki67 index | | | <0.001 | | | <0.001 |
| Low | 248 (71.1) | 53 (30.3) | | 214 (76.4) | 87 (35.7) | |
| High | 101 (28.9) | 122 (69.7) | | 66 (23.6) | 157 (64.3) | |
| P53 overexpression | | | <0.001 | | | <0.001 |
| Absent | 286 (81.9) | 100 (57.1) | | 232 (82.9) | 154 (63.1) | |
| Present | 63 (18.1) | 75 (42.9) | | 48 (17.1) | 90 (36.9) | |
| Subtype | | | <0.001 | | | <0.001 |
| Luminal A | 195 (55.9) | 30 (17.1) | | 169(60.4) | 56 (23.0) | |
| Luminal B | 96 (27.5) | 43 (24.6) | | 71 (25.4) | 68 (27.9) | |
| HER2+ | 19 (5.4) | 45 (25.7) | | 16 (5.7) | 48 (19.7) | |
| Triple negative | 39 (11.2) | 57 (33.4) | | 24 (8.6) | 72 (29.5) | |

*P* values are calculated by Chi-square or Fisher's exact test. Number in parenthesis indicates percentage.

TC, tumor cell; IC, immune cell; LVI, lymphovascular invasion; ER, estrogen receptor; PR, progesterone receptor

**Supplementary Table S5. Correlations between S100A8+ immune cells, infiltration of CD4+, CD8+, and FOXP3+ tumor infiltrating lymphocytes and PD-L1+ immune cells in pre-invasive and invasive carcinoma**

| Disease group | Correlation between markers | S100A8+ IC | CD4+ TIL | CD8+ TIL | FOXP3+ TIL | PD-L1+ IC |
|---|---|---|---|---|---|---|
| Pre-invasive carcinoma | S100A8+ IC | - | 0.281 (<0.001) | 0.249 (0.001) | 0.209 (0.006) | 0.248 (0.001) |
| | CD4+ TIL | 0.281 (<0.001) | - | 0.610 (<0.001) | 0.463 (<0.001) | 0.391 (<0.001) |
| | CD8+ TIL | 0.249 (0.001) | 0.610 (<0.001) | - | 0.458 (<0.001) | 0.344 (<0.001) |
| | FOXP3+ TIL | 0.209 (0.006) | 0.463 (<0.001) | 0.458 (<0.001) | - | 0.434 (<0.001) |
| | PD-L1+ IC | 0.248 (0.001) | 0.391 (<0.001) | 0.344 (<0.001) | 0.434 (<0.001) | - |
| Invasive carcinoma | S100A8+ IC | - | 0.263 (<0.001) | 0.474 (<0.001) | 0.482 (<0.001) | 0.525 (<0.001) |
| | CD4+ TIL | 0.263 (<0.001) | - | 0.487 (<0.001) | 0.306 (<0.001) | 0.336 (<0.001) |
| | CD8+ TIL | 0.474 (<0.001) | 0.487 (<0.001) | - | 0.588 (<0.001) | 0.616 (<0.001) |
| | FOXP3+ TIL | 0.482 (<0.001) | 0.306 (<0.001) | 0.588 (<0.001) | - | 0.565 (<0.001) |
| | PD-L1+ IC | 0.525 (<0.001) | 0.336 (<0.001) | 0.616 (<0.001) | 0.565 (<0.001) | - |

Data are presented as rho correlation coefficients calculated by Spearman's rank correlation test and p values in parentheses.

TIL, tumor-infiltrating lymphocyte; IC, immune cell
